# Cystadenofibroma and contralateral collision lesions: A unique ovarian case report

**DOI:** 10.18632/oncoscience.616

**Published:** 2025-03-31

**Authors:** Naina Kumar, Ashutosh Rath, Aparna Setty, Pooja T. Rathod, Jarathi Aparna

**Affiliations:** ^1^Department of Obstetrics and Gynecology, All India Institute of Medical Sciences, Bibinagar, Hyderabad, Telangana 508126, India; ^2^Department of Pathology, All India Institute of Medical Sciences, Bibinagar, Hyderabad, Telangana 508126, India

**Keywords:** collision tumor, cystadenofibroma, mucinous, ovary, serous

## Abstract

Introduction: Ovarian cystadenofibromas are rare benign tumors, accounting for only 1.7% of benign ovarian neoplasms. Even rarer are ovarian collision tumors, with the coexistence of collision tumors and other benign ovarian neoplasms being exceptionally uncommon. This report presents a unique case of serous cystadenofibroma in one ovary, accompanied by collision features involving serous and mucinous cysts in the contralateral ovary. Case report: A 41-year-old woman presented with lower abdominal pain and swelling persisting for 2–3 months. Clinical evaluation of the abdomen revealed a mobile, non-tender, cystic mass resembling a 26–28-week gravid uterus, with no free fluid. Local and per speculum examinations showed a healthy vulva, cervix, and vagina, with a Pap smear negative for intraepithelial lesions or malignancy. A bimanual examination identified a mobile, multiparous uterus and a large (~15 × 12 cm), predominantly cystic lesion originating from the right adnexa and extending anteriorly and superiorly to the uterus. MRI findings confirmed these observations. Given the endometrial biopsy indicating endometrial intraepithelial neoplasia, the patient underwent an exploratory laparotomy with total abdominal hysterectomy and bilateral salpingo-oophorectomy. Histopathological analysis revealed serous cystadenofibroma in the right ovary and multiple serous and mucinous cysts in the left ovary, consistent with collision features. Additionally, the uterine endometrium showed hyperplasia without atypia. Conclusion: This case underscores the rare coexistence of a benign surface epithelial-stromal tumor in one ovary and collision features in the other. It emphasizes the importance of comprehensive evaluation, precise diagnosis, and timely surgical management to ensure optimal patient outcomes.

## INTRODUCTION

Serous cystadenofibromas are rare benign ovarian surface epithelial-stromal tumors, accounting for approximately 1.7% of benign ovarian neoplasms [1, 2]. These tumors comprise dense fibrous stroma interspersed with epithelial cystic components [3]. They are classified into serous, endometrioid, mucinous, clear-cell, and mixed subtypes, with the serous subtype being the most common, representing approximately 75% of cases [4]. Typically, unilateral, serous cystadenofibromas are most frequently observed in women aged 15–65 years [1]. Clinically, these tumors are often asymptomatic and are incidentally discovered during routine gynecological ultrasounds. However, when symptoms do manifest, they may include lower abdominal pain, vaginal bleeding, or a palpable abdominal mass [4, 5]. Occasionally, symptoms such as metrorrhagia or signs of feminization may occur, likely due to tumor-induced hyperestrogenism. This hyperestrogenic state is thought to result from excessive hormone production directly associated with the tumor [6].

Collision tumors, on the other hand, are rare neoplasms characterized by the coexistence of two distinct tumors within the same organ without histological intermingling [7]. Although collision tumors can occur in various organs such as the stomach, liver, and lungs, their presence in the ovary is uncommon. Among ovarian collision tumors, teratomas are among the most frequent components observed. These tumors are typically unilateral, with sizes ranging from 5.5 to 200 cm, and are most commonly found in patients aged 17 to 66 years [8, 9]. Each component of a collision tumor develops independently without any direct connection, and its unique characteristics dictate its biological behavior [9]. The clinical presentation is often nonspecific, with many patients remaining asymptomatic. Some individuals, however, report symptoms such as intermittent abdominal pain, bloating, or frequent urination, which are typically due to the compression of adjacent structures by the tumor mass [10]. Rarely, symptoms such as ascites or abnormal uterine bleeding may also occur [11]. The biological behavior of these tumors is determined by the nature of their components. If one of the tumor components is malignant, it may exhibit aggressive features, including local tissue invasion, involvement of adjacent organs, and distant metastasis [10, 12].

This case report highlights a rare occurrence of serous cystadenofibroma in one ovary alongside collision features involving serous and mucinous cysts in the contralateral ovary. Such findings are exceptionally rare in the literature, emphasizing the importance of documenting and reporting these unique cases to enhance the understanding of their pathogenesis and clinical presentation.

## CASE REPORT

A 41-year-old woman, para 3 live 2, presented to the Obstetrics and Gynecology outpatient department with a 2–3-month history of lower abdominal pain and swelling. She denied symptoms such as vomiting, appetite loss, weight loss, irregular menstrual cycles, dysmenorrhea, or heavy menstrual bleeding. Her last menstrual period was on November 18, with a regular menstrual cycle of 28–30 days and 2–3 days of bleeding. She had no significant medical history.

On examination, her body mass index (BMI) was 23.8 kg/m², and her vital signs were normal. Abdominal examination revealed a mobile, non-tender, cystic mass resembling a 26–28 weeks gravid uterus, with no free fluid detected. Local and per speculum examinations showed a healthy vulva, cervix, and vagina, with a negative Pap smear for intraepithelial lesions or malignancy. Bimanual examination revealed a mobile multiparous uterus and a large, predominantly cystic lesion (~15 × 12 cm) originating from the right adnexa, extending anteriorly and superiorly to the uterus. The left ovary was also enlarged and palpable.

**Figure 1:**
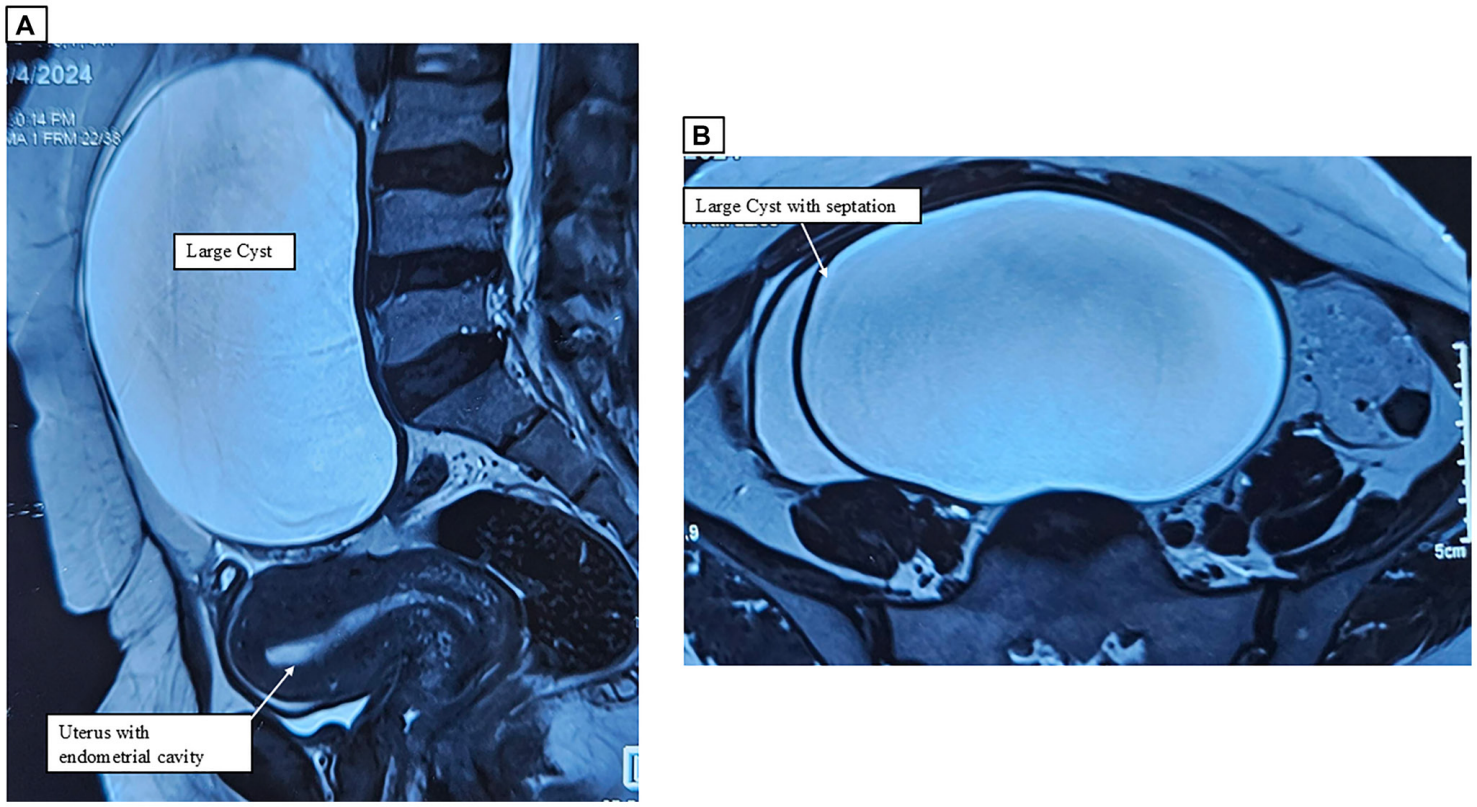
(**A, B**) MRI showing a large multiloculated cystic lesion with normal uterus and left ovary.

Abdominal ultrasonography showed a 68 × 44 × 30 mm uterus with an 8.9 mm endometrial thickness (ET) and a 14.3 × 11.8 × 13 cm anechoic cystic lesion in the right adnexa, compressing the bladder, suggestive of a complex ovarian cyst (serous/mucinous cystadenoma). The left ovary appeared normal. Tumor markers revealed CA-125 levels of 22.3 U/ml, HE4 levels of 52.7 pmol/L, and a Risk of Ovarian Malignancy Algorithm (ROMA) score of 21.6%. Magnetic Resonance Imaging (MRI) confirmed a multilocular cystic lesion (~14 × 14 × 8.2 cm) arising from the right ovary (ORADS III), with mild ascites and no significant lymphadenopathy (Figure 1A, 1B).

**Figure 2:**
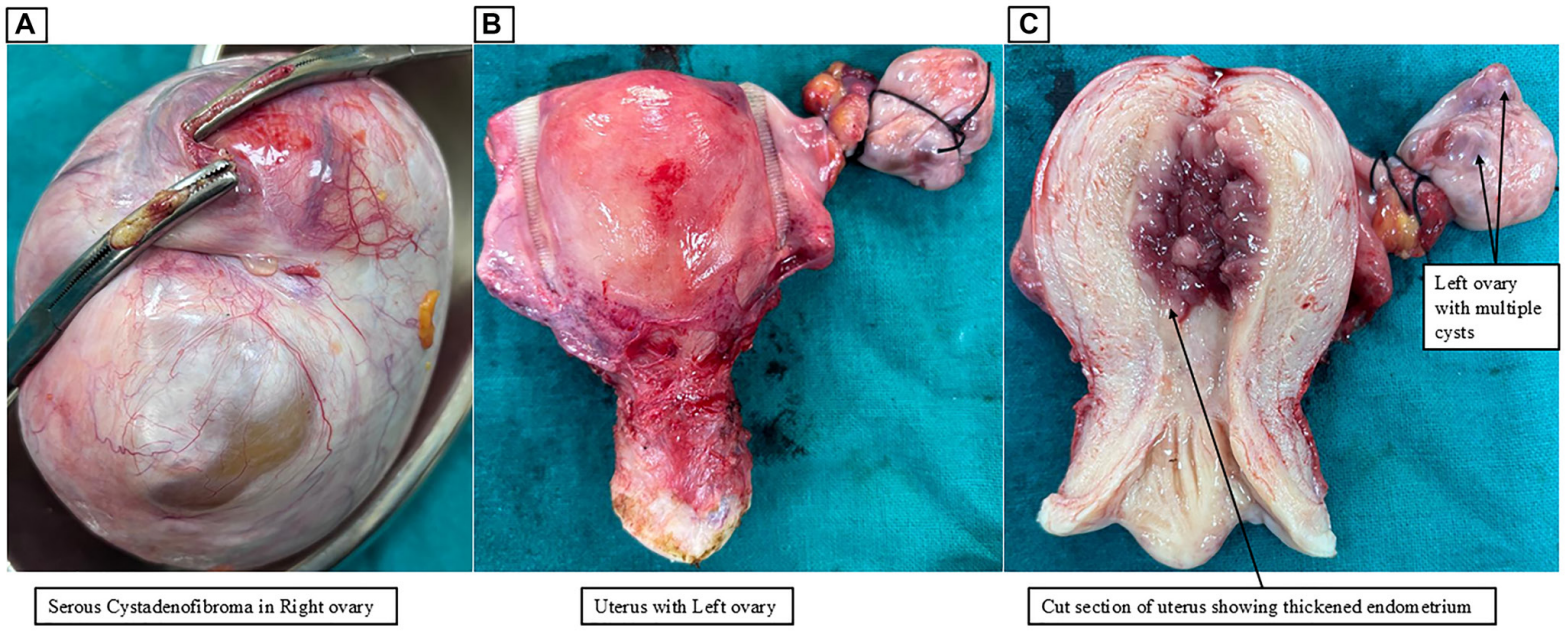
(**A**) Gross appearance of the right ovarian serous cystadenofibroma; (**B**) Gross appearance of the uterus and left ovary; (**C**) Cut open appearance of the uterus with thickened endometrium and left ovary with multiple cysts.

Anemia correction with two units of blood transfusion was performed (initial hemoglobin 7.5 g/dL). An endometrial biopsy revealed endometrial intraepithelial neoplasia (EIN). Following anemia correction, the patient underwent exploratory laparotomy with total abdominal hysterectomy and bilateral salpingo-oophorectomy. Intraoperatively, the right ovary had a 14 × 15 cm predominantly cystic lesion extending over the uterus, while the left ovary exhibited 2–3 cystic lesions (largest ~3 × 4 cm) (Figure 2A–2C). Minimal ascites (~100–150 ml) were noted, with cytology negative for malignancy. Other intra-abdominal structures, including the omentum, peritoneum, intestines, liver, and diaphragm, appeared grossly normal.

**Figure 3:**
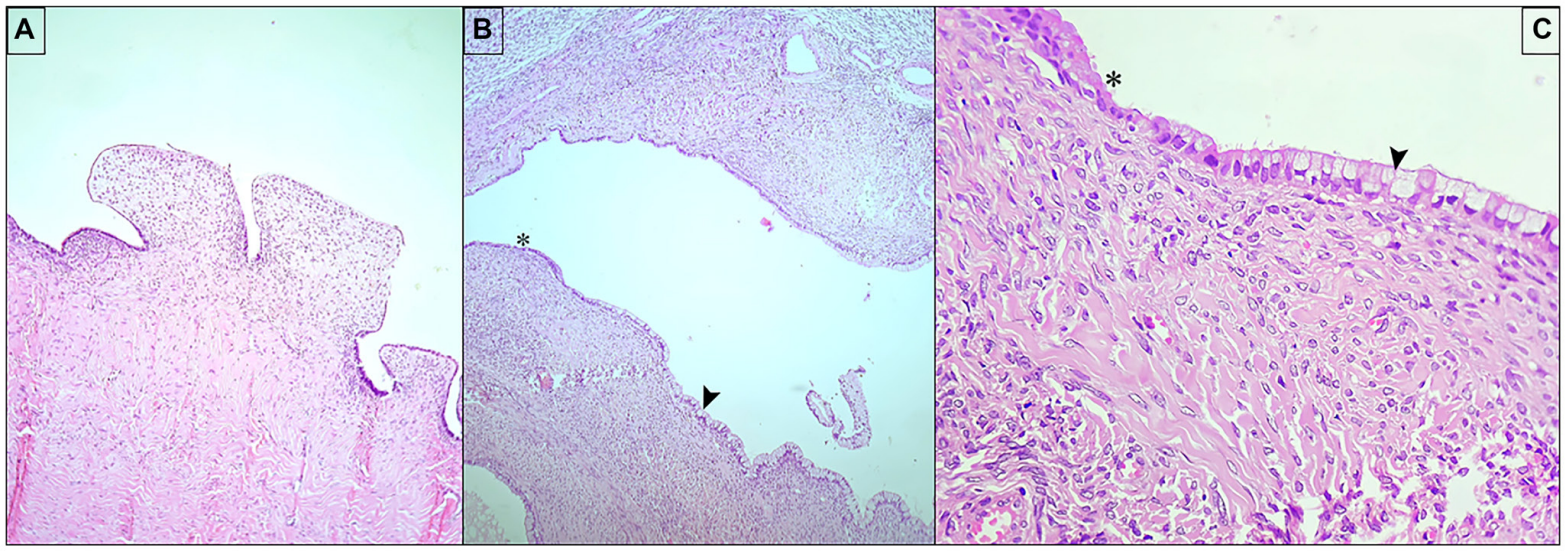
(**A**) Serous cystadenofibroma, right ovary (10×); (**B**, **C**) Sero-mucinous cystic follicles, left ovary, lined by serous (^*^) and mucinous (arrow) lining epithelium (3b −10×; 3c −40×).

Gross pathology revealed the right ovary (14 × 13 × 8 cm) with two cystic cavities filled with serous fluid and thin walls (0.2 cm) with papillary excrescences. The left ovary (4.5 × 3.5 × 4 cm) contained multiple cysts, the largest measuring 3.5 cm. Microscopic examination confirmed serous cystadenofibroma in the right ovary and multiple serous and mucinous cysts in the left ovary, indicative of collision features (Figure 3A–3C). The uterus exhibited hyperplasia without atypia, while the cervix showed chronic cervicitis. Bilateral fallopian tubes, omentum, and peritoneum were histologically unremarkable.

Histopathological analysis confirmed the rare coexistence of serous cystadenofibroma in one ovary and collision features in the contralateral ovary. The patient tolerated the surgery well, with an uneventful postoperative recovery. Sutures were removed, and she was discharged in stable condition on the eighth postoperative day.

## DISCUSSION

Surface epithelial-stromal tumors of the ovary are classified into five primary subtypes: serous, mucinous, endometrioid, clear cell, and transitional cell, with the possibility of mixed or combined forms among these categories [13]. According to the World Health Organization (WHO) classification, benign ovarian tumors encompass a diverse range of entities, including papillomas, cystadenomas, adenofibromas, cystadenofibromas, metaplastic papillary tumors, and endometrioid polyps. Cystadenofibromas, specifically, originate from invaginations of the ovarian surface epithelium, a process linked to the natural morphological changes that occur in the ovary during the reproductive years. The fibrous component of these tumors results from varying degrees of cortical stromal proliferation and collagen deposition [14]. Based on the degree of epithelial proliferation, cystadenofibromas are categorized as benign, borderline, or malignant, although malignant transformation is exceedingly rare [4].

These tumors exhibit three primary morphological patterns on imaging: multilocular solid lesions with a “black sponge” appearance, unilocular cystic lesions with parietal thickening, and purely cystic masses. Imaging features, such as T2-weighted hypointensity (referred to as the “dark-dark appearance”), absence of diffusion restriction, and progressive post-contrast enhancement (type I perfusion curve), are characteristic. However, these features can lead to misdiagnosis as malignancies due to their solid components or irregular septations [4, 6, 15]. Tumors that often mimic ovarian cystadenofibromas include fibromas, Brenner tumors, struma ovarii, highly fibrous metastatic ovarian tumors (especially from the gastrointestinal tract), and endometriomas [4, 16]. Furthermore, the other differentials for bilateral ovarian masses include Krukenberg’s tumor [17], ovarian tuberculosis [18, 19], endometriomas [20], and very rarely polycystic ovarian disease [21].

Macroscopically, serous cystadenofibromas vary significantly in size, ranging from an average of 5–8 cm to as large as 30 cm in diameter. Larger tumors pose a notable risk for ovarian torsion, particularly due to their ability to grow substantially [16]. Accurate diagnosis requires a combination of imaging and histopathological correlation. Complete surgical excision is the preferred treatment, with most cases yielding an excellent prognosis when managed appropriately [5].

In contrast, ovarian collision tumors consist of epithelial, germ cell, and sex-cord-stromal tumor combinations [12]. The most common combination involves mucinous cystadenoma coexisting with other tumor types, such as Brenner tumor, mature cystic teratoma, Sertoli-Leydig cell tumor, or serous cystadenoma [22, 23]. The pathogenesis of collision tumors remains unclear, with proposed hypotheses ranging from the simultaneous proliferation of distinct cell lines to differentiation from a common pluripotent precursor stem cell [9]. Other theories suggest mechanisms such as [24–26]:

- *Chance Occurrence:* Collision tumors may arise from the coincidental, simultaneous proliferation of two distinct cell lines (“chance accidental meeting”).
- *Microenvironmental Influence:* The presence of the first tumor could alter the local microenvironment—through mechanisms such as angiogenesis and inflammation—promoting the development of a second primary tumor or facilitating the seeding of metastatic cells.
- *Carcinogenic Exposure:* A carcinogenic agent interacting with different tissues may induce the formation of distinct tumors.
- *Proximity of Origin:* The occurrence of two different histological subtypes may be a result of the tumors arising independently from neighboring tissues.

While none of these theories fully explain all cases, they offer insight into the complex mechanisms that may contribute to the development of ovarian collision tumors [24, 25].

Seromucinous neoplasms, a newly recognized category of ovarian epithelial tumors introduced in the 2014 revised WHO classification, further expand the spectrum of ovarian tumors. These rare tumors, derived from Müllerian tissue, are classified into benign, borderline, and malignant categories. They are characterized by a unique combination of cell types, including endocervical-type mucinous, endometrioid, and squamous epithelial elements [13].

Recent case reports have documented the coexistence of serous and mucinous cystadenomas within a single ovary, underscoring the rarity of such findings [23].

Most collision tumors are typically diagnosed postoperatively through histopathological examination. Currently, there are no standardized management protocols for collision tumors, and treatment is largely guided by the tumor’s specific components. In clinical practice, both management strategies and prognosis depend on several key factors, including the histological types of the colliding components, the most aggressive component present, and the stage of the malignant tumor [9, 27]. Benign tumors are generally managed with surgical excision alone, while malignant components often require a combination of surgery, radiation therapy, and chemotherapy. Pelvic and para-aortic lymph node dissection is not recommended for patients with benign ovarian tumors. Furthermore, studies indicate that in early-stage ovarian cancer, lymph node dissection does not significantly improve progression-free or overall survival rates and may contribute to increased postoperative morbidity [28–30]. In the present case, preoperative evaluation—including clinical assessment, radiological imaging, tumor marker analysis, and intraoperative findings—strongly suggested the benign nature of the ovarian masses, thereby obviating the need for pelvic lymph node dissection. Furthermore, the frozen section of the masses was not performed, as the endometrial biopsy revealed EIN, and the patient expressed a preference against conservative management. Consequently, a total abdominal hysterectomy with bilateral salpingo-oophorectomy was undertaken. Accurate preoperative diagnosis is critical for effective treatment planning and avoiding unnecessary interventions that could pose additional risks to the patient [10].

Table 1 summarizes previously reported ovarian collision tumors with diverse histological components [7, 22, 25, 26, 31–35].

**Table 1:** Rare ovarian collision tumors with diverse histological components

Author and Year	Patient’s age and presenting complaints	Radiological findings	Histopathological findings	Remark	References
Shopov, 2020	A 74-year-old postmenopausal woman presented with lower abdominal pain	Transvaginal sonography revealed a polycystic left pelvic mass measuring 11.5 cm with a hyper-echoic sector.	Serous cystadenoma and mixed hemangioma with stromal luteinization in the left ovary.	An exceptionally rare ovarian collision tumor with only a few cases reported in the literature.	[31]
Mongelli, et al., 202 2	A 74-year-old postmenopausal woman presented with sudden onset lymphoedema in the right leg	Ultrasound examination revealed a unilocular cyst with solid low-level echoes, measuring 128 × 65 mm in the left ovary.	Collision tumor consisting of Fibrothecoma and serous cystadenoma in the left ovary.	The presence of both neoplastic Müllerian epithelial and sex cord-stromal components within a single tumor is exceptionally rare.	[32]
Jacob, et al., 2022	A 40-year-old nullipara presented with lower abdominal pain and fullness associated with vomiting.	Transabdominal ultrasound revealed a dermoid component with cystic areas, echogenic thick walls, and a linear hyperechoic area measuring 13.2 × 8.3 cm in the right ovary.	Right ovarian collision tumor consisting of mature cystic teratoma and ovarian fibroma complicated by torsion.	Collision tumors involving a mature cystic teratoma and ovarian fibroma are uncommon.	[26]
Mukilarasi, et al., 2023	A 39-year-old woman presented with abdominal distension and abdominal discomfort.	Abdominal and transvaginal ultrasound revealed a cystic mass measuring 230 mm × 180 mm × 120 mm in right ovary	Collision tumor of right ovary comprising of mature cystic teratoma and Mucinous cystadenoma.	A collision tumor composed of a mucinous cystadenoma and a mature cystic teratoma is the most frequently reported type.	[25]
Balhara, et al., 2023	A 56-year-old postmenopausal woman presented with complaints of left lower abdominal pain.	MRI pelvis revealed a large cystic space occupying lesion measuring 132 mm × 102 mm in the left adnexa.	Collision tumor of left ovary consisting of serous cystadenoma and fibroma.	The coexistence of surface epithelial tumors and those from the thecomafibroma group is exceptionally rare.	[22]
Omo-Ogboi, et al., 2023	A 60-year-old postmenopausal woman presented with pain in the abdomen.	CT revealed a large soli-cystic lesion in the left adnexa.	Collision tumor composed of high-grade serous carcinoma and Sertoli-Leydig cell tumor	The coexistence of ovarian high-grade serous carcinoma and a Sertoli-Leydig cell tumor is exceptionally rare.	[33]
Rjoop, et al., 2024	A 60-year-old woman presented with post-menopausal bleeding and abdominal pain.	CT revealed a solid cystic lesion in the right adnexa.	Ovarian collision tumor consisting of fibroma and serous cystadenoma in right ovary with small serous cystadenoma in left ovary.	Only seven cases of this particular combination of ovarian tumors have been reported in the literature.	[7]
Liu, et al., 2024	A 73-year-old woman presented with abdominal distension and pain.	Pelvic ultrasound revealed a benign ovarian cyst of 14.2 cm × 8.3 cm × 6.9 cm in size along with leiomyoma. CT and MRI revealed a right ovarian malignant tumor.	Right mixed ovarian tumor (thecoma-fibroma, 70%; serous cystadenoma, 20%; and diffuse large B-cell lymphoma, 10%) and left ovarian thecoma-fibroma.	A rare ovarian collision tumor comprising a thecoma-fibroma, serous cystadenoma, and diffuse large B-cell lymphoma, with only a few cases reported in the literature.	[34]
Lee, et al., 2024	A 63-year-old postmenopausal woman presented with abdominal discomfort.	MRI revealed a multiloculated cyst (11.7 cm) with a watery fluid or internal hemorrhage in the left ovary.	Left ovarian collision tumor composed of an adult granulosa cell tumor and mesonephric-like adenocarcinoma.	Such tumors reflect the coexistence of both indolent and aggressive tumor components in the ovary.	[35]

## CONCLUSIONS

This case underscores the rare coexistence of serous cystadenofibroma in one ovary and collision features involving serous and mucinous cysts in the contralateral ovary, a combination scarcely reported in the literature. Ovarian collision tumors, characterized by distinct tumor types without histological intermixing, and cystadenofibromas, which are benign epithelial-stromal tumors, both represent diagnostic challenges due to their variable clinical presentations and imaging features. The importance of comprehensive preoperative evaluation, including imaging and tumor marker analysis, is evident in ensuring accurate diagnosis and optimal management. Histopathological examination remains the cornerstone of definitive diagnosis, differentiating between benign and malignant components to guide appropriate treatment. In this case, surgical intervention successfully managed the lesions, resulting in an excellent prognosis.
